# Moving toward a process-oriented perspective in the personalized treatment of depression

**DOI:** 10.1007/s00406-021-01249-9

**Published:** 2021-03-05

**Authors:** Johannes Kopf-Beck, Julia Fietz

**Affiliations:** 1 Department of Translational Research in Psychiatry, Max Planck Institute of Psychiatry, Kraepelinstraße 2-10, 80804 Munich, Germany; 2 International Max Planck Research School for Translational Psychiatry (IMPRS-TP), Munich, Germany

Research into the underlying parameters and mechanisms involved in the emergence and maintenance of major depressive disorder (MDD) has elicited the development of new treatment approaches and improved existing ones, for instance, pharmacotherapy or psychotherapy. Even though there has been significant progress on different levels and in different fields, the efforts to gain a better understanding of MDD have faced constant challenges. High relapse rates and lack of treatment responsiveness leave much room for improvement and call for new insights to reduce the disease burden in MDD—for affected individuals as well as for societies.

Regarding the development of new treatments of depression, ketamine and esketamine, represent such an approach from the pharmacological field and has a rapid anti-depressive effect. In the current issue of European Archives of Psychiatry and Clinical Neuroscience, Leal and colleagues present results from an open-label pilot study [1] showing first evidence for positive effects of arketamine on depressive symptoms in persistent forms of depression. Thus, by confirming findings from animal studies, they provide a platform upon which future prospective randomized controlled trials (RCTs) can be built to further pursue this translational strategy. Kaur et al. [2] review the current research status of esketamine in treatment-resistant depression and conclude that it could serve as an important contribution to a multi-modal approach when combined with oral anti-depressant medication (ADM), not least due to its rapid response, bridging the gap between initiating an established ADM and the onset of patient response.

The development of new treatment modalities, such as ketamine, and the alteration of existing ones to better target-specific symptom profiles emphasizes the need for individualized pharmacological and psychotherapeutic care in MDD. Establishing criteria for optimized treatment allocation remain the goal of extensive research.

Within this context, Imai et al. [3] implement a symptom-based strategy to predict treatment response by analyzing individual patient data from three RCTs. They show that although melancholic features predict treatment outcome, they do not moderate the effect of ADM over placebo, and as such the authors do not recommend its use as a criterion for ADM treatment decisions. Further, a recent metanalysis on the limited predictive value of individual symptom characteristics in the context of treatment choice between ADM and psychotherapy for MDD [4] suggests that we need to shift our focus to the underlying and transdiagnostic processes. This might constitute a more promising approach in predicting the therapeutic value of specific interventions.

The Research Domain Criteria (RDoC) approach aims to establish the classification of mental disorders based on neurobiological processes and integrate them with the associated behavioral correlates, thus providing a transdiagnostic, dimensional and process-oriented perspective [5]. The domains *negative* and *positive valence*, *cognitive systems*, *social processes*, *arousal and regulatory systems* and *sensorimotor systems* do not only represent a diagnostic framework beyond symptoms, but also the basis for the development of process-oriented treatments.

Two contributions in the current volume pursue the RDoC-perspective. Chen et al. [6] investigated the role of working memory (WM), a construct of the cognitive systems domain within the RDoC framework, during repeated ketamine treatment in MDD patients. They find that enhanced WM function relates to the positive effect of ketamine on suicidal ideation and can potentially predict anti-suicidal response after six ketamine infusions. Surova and colleagues [7] present another RDoC-based approach using levels of brain arousal as criteria to reveal different clinically relevant subgroups in a population of MDD patients with fatigue. Here, they identify higher levels of concentration difficulties, lack of energy, and sleepiness in the hyperaroused group, and more suicidal thoughts in the non-hyperaroused group. Such findings represent an important step toward a deeper understanding of the neuropsychological mechanisms driving these disorders and toward treatments guided by psychopathological processes: a worthwhile consideration in clinical practice.

Nevertheless, previous research on the effectiveness of different types of MDD treatments, such as ADM or psychotherapy, have often relied upon general outcome parameters, ignoring the underlying processes and their temporal dynamics. This applies especially to psychotherapy research, where RCTs on treatment protocols for disorders have dominated the research landscape. Unsatisfactory answers regarding the superiority of a certain approach and the question “What works for whom?” have spurred on the differentiation of psychotherapeutic approaches.

In particular, cognitive behavioral therapy (CBT) underwent this differentiating development, for example, with the establishment of mindfulness-based CBT, schema therapy, and the Cognitive Behavioral System of Psychotherapy (CBASP), which specifically targets the demands of patients suffering from persistent depressive disorder (PDD).

CBASP focuses on biographical experiences of maltreatment during childhood and the resulting interpersonal deficits, which can be assigned to the RDoC-domain of social processes [5]. In a naturalistic study, Guhn and colleagues [8] adopt a 12-week CBASP treatment protocol adapted to the needs of PDD patients, who are typically represented in acute psychiatric inpatient settings. The authors show large effect sizes in clinical ratings as well as in self-reports regarding the decrease of depressive symptoms, both immediately after treatment and at follow-up.

Such therapy approaches, which tailor down broad, disorder-based treatments to particular target groups, recognize specific processes, such as interpersonal deficits, and consider their relationship with symptom severity. Thus, they take an important step toward a more process-based understanding of the psychopathology of MDD and its treatment.

Further translation of a process-oriented, dimensional, and transdiagnostic perspective of the RDoC approach into the behavioral sphere would open up new avenues for the development of psychotherapy treatments. It could be a promising strategy to link RDoC-constructs, such as perception and understanding of self and others, cognitive control, arousal, or reward learning, with their corresponding psychological and behavioral counterparts, such as cognitive biases, emotion dysregulation, lack of motivation, or lack of reinforcing environments. These psychological and behavioral correlates could represent the target of specific intervention modules, which could be applied in personalized psychotherapy approaches rather than as a comprehensive treatment protocol for a heterogenous disorder such as MDD. Such a link not only allows for a more precisely fitting therapy, but also closes the gap between clinically relevant phenotypes and research on underlying RDoC-dimensions.

Measuring the outcome of such interventions modules provides an opportunity to overcome prior shortcomings in psychotherapy research, which was often based on the effects of broad treatments on a broad range of symptoms or even questionnaire sum score levels. The idea of capturing the effects on a process level, ranging from molecular manifestations to objective behavioral markers and subjective self-reports, and accounting for process dynamics over time, for example, by ecological momentary assessment, will ensure more precise insights into what drives depression and how to address it best.

By integrating a mechanism-based understanding of psychopathology and process-based psychotherapy [9], we can develop and validate accurate interventions, thereby bringing us one step closer toward the goal of personalized psychiatry.
